# The Uses and Abuses of Cocaine, with Reference to Mucous Membranes Especially*Read at the tenth annual meeting of the American Rhinological Association, at Indianapolis, September 22, 1892.

**Published:** 1892-11

**Authors:** Arthur G. Hobbs

**Affiliations:** Atlanta, Ga.; Ex-President of the American Rhinological Association, Member of the American Medical Association, of the Georgia State Medical Association, the Atlanta Society of Medicine, etc., etc.


Southern Medical Record.

A MONTHLY JOURNAL OF MEDICINE AND SURGERY.

Vol. XXII. Atlanta, Ga., November, 1892. No. 11

### Original Articles.

### THE USES AND ABUSES OF COCAINE, WITH REFERENCE TO MUCOUS MEMBRANES ESPECIALLY.*

BY ARTHUR G. HOBBS, M. D., ATLANTA, GA.

Ex-President of the American Rhinological Association, Member of the American Medical Association, of the Georgia State Medical Association, the Atlanta Society of Medicine, etc., etc.

Since I received my first twenty grains of the hydrochlorate of cocaine (a part of the first two-drachm package that arrived in New York), I have used many ounces; perhaps, altogether, by myself and assistants, nearly as many pounds as myoriginal package contained grains.

It will not be amiss for me to say in the beginning, that I now have very different opinions of its uses from those I entertained during the first years of its advent. However, one of my original opinions of its applicability has only become more and more confirmed with my increased use of it: and that is that its utility is principally confined to its local application to mucous membranes.

*Read at the tenth annual meeting of the American Rhinological Association, at Indianapolis, September 22, 1892.

The softer the mucous tissue to which it is applied, the sooner is its effect reached and the less the quantity necessary; and, as a consequence, the quicker and more decided is the toxic effect when its application is persisted in to this extent.

The general symptoms, however, are, everything else being equal, much oftener present in proportion to the area of membrane surface involved, than in proportion to the strength of the solution applied. Hence, sprays of a weak solution are liable to produce toxic symptoms, when much stronger solutions can be applied with a saturated cotton probe or wad, to a limited area, almost with impunity.

The constitutional effects of cocaine are manifested by a feeling of faintness—it may be even to the loss of consciousness—a trembling of the limbs, a pallor of the face, etc., any or all of which may appear in one minute or in fifteen minutes after the application to a large surface. Again, these symptoms may be produced by a small quantity of a one per cent. solution in one individual and not in another, even if in the latter both the strength and quantity be ten times multiplied. I have never yet reached a conclusion as to the average amount of cocaine that is necessary to produce the constitutional symptoms, neither do I ever know, the first time I use it, how to estimate the individual's susceptibilities.

Every individual seems to be a law unto himself as to the quantity necessary in his own case to accomplish complete local anæsthesia, and even in the same person the amount will vary at different times with no apparent cause. Under similar circumstances I find it impossible to reach a complete local anæsthesia in some, short of the toxic symptoms; when in others, one-tenth of the amount and one-tenth of the time will render the application of a galvano-cautery absolutely painless.

To obviate the unpleasantness and delay caused by the constitutional symptoms it is my custom to administer a stimulant of brandy or whisky at their first indications, and make the operation quickly and, always under such circumstances, painlessly. When it is found by trial that one bears the drug badly, it would be well always to give a stimulant before the second application is made.

I am sure that the last thousand galvano-cautery operations I have made would represent a much greater per cent. that were absolutely painless than the nearly three thousand cauteries that preceded. Perhaps this has been due to my greater familiarity with the technique of cocaine applications in the more recent cases.

In some its effects in the superior pharynx are peculiarly distressing, more especially at the first application, but less and less so afterwards. However, it is well to remember this and avoid it, when making local applications above, by not allowing the solution to gravitate from the cotton plug.

The time necessary for cocainizing a mucous membrane is perhaps as indefinite as the strength of the solution, both due to that unknown quantity, the individual. Approximately, however, four to ten minutes and a four to a ten per cent. solution for a small surface may be regarded as an average. If the intention is to produce the effect over a small surface only, as in galvano-cautery operations, a concentrated solution—ten to twenty per cent—should be used and the result expected in four to eight minutes.

If, however, a general turgescence of any membrane should suggest the use of cocaine, a spray of a very weak solution—a one or a two per cent—should be expected to accomplish the desired result in one or two minutes. In either case, the general effects may appear without any warning and mar the anticipated good results, or at least, render an otherwise easy operation, tedious and unpleasant.

It has been my custom from the beginning to make my solutions at the moment I use them and to vary their strength according to the case, and as I use no weights or measures I never know, only approximately, the strength. The range is so great that I hesitate in stating it as I have not space to give my reasons for sometimes using a one grain to the minim solution, and again, only a one grain to five hundred minims. As a general rule, however, the strength should be in inverse proportion to the surface to which it is applied. Since my many disappointments with ready-made solutions of cocaine, during the first few months of its advent I have never risked any solution that had not been recently dissolved.

In case it should be necessary to dissolve a large amount, it would be well to add boric acid for its preservation, in a proportion of about one grain of the latter to ten grains of the former.

For safety, it is best to supply the drug instead of giving the patient a prescription to have filled and bring with him for an appointed operation. The operation will thus be less magnified in the patient's mind, and he will be less liable to familiarize himself with the proportions and uses of the drug, should the temptation to use it himself arise afterwards. For these reasons, I do not, in any instance, give a patient a prescription containing cocaine as its leading ingredient.

As an ingredient in sprays, I find it abused more, perhaps, in hay fever prescriptions than anywhere else. This is true also where it is prescribed in the form of powders for this disease.

The turgescent condition of all the mucous membranes of the upper respiratory tract, especially in the nasal turbinates, that is always present in hay fever patients, is so quickly palliated and rendered bearable, that the temptation is great to prescribe cocaine in some form for the patient's own use; but it is a dangerous experiment, and one to be ventured upon only in exceptional cases.

During the past few years I have seen many journal articles that claimed for it a decided aphrodisiac effect, while my observations have been universally to the contrary. It will not be departing too far from the subject if I mention the effects of cocaine (through the mucous membranes) upon two cases of priapism, notwithstanding the fact that the results in these cases were reached through the general systemic impressions of the drug. The two young men, who were the victims of this rather unusual disorder, came under my observation by accident, and at intervals of about three years; each came to my office in a carriage for a coincident nasal and pharyngeal inflammation. Cocaine dissolved in vaseline, together with a small quantity of campho-mentholine, was sprayed into the nasal and pharyngeal cavities, to the great relief of all the unpleasant symptoms in these regions.

But this was not all. In the first case, to my surprise and to the patient's great joy, the other and more painful malady disappeared within ten minutes. In the second case, the symptoms were almost exactly like the first, and the patient's delight was greater than my surprise, as I was this time anticipating a similar result from the same treatment. The relief of the priapism in both cases remained permanent.

In both instances, the usual treatment for such cases had been resorted to apparently without any result for twenty-four and thirty-six hours respectively, only to be completely relieved by a cocaine spray used upon a distant mucous membrane for a coincident malady. It would seem that in these cases cocaine acted as an anaphrodisiac, and it should not be surprising when we remember that its primary effect is to reduce a turgescence of the turbinates, and that these tissues are analogous in many respects to the erectile tissues that are involved in priapism.

I desire here to emphasize most decidedly one contra-indication in the use of cocaine as a collyrium, and that is that *it should never be used when an abrasion of the cornea exists;* nothing in the form of a collyrium is more deceptive, as it is also most grateful to a denuded corneal surface.

The cornea is covered with the epithelial layer of the conjunctival mucous membrane only; here no middle layer proper exists whence it can derive its nutrition, hence it quickly loses its vitality when subjected to this local anæsthetic. So also, the outer layer of the cornea, receiving its nutrition by imbibition only, seems to be even more susceptible to the destructive effects of cocaine.

I have seen a number of cases, during the last few years, (especially since this drug has been introduced into general use), in which the cornea has been greatly damaged by its constant and frequent introduction, for no other purpose than the temporary relief of pain; but unfortunately the relief thus produced proves to be a delusion, and the destruction produced upon the cornea is only masked for the time by the anæsthetic effects.

I have especially seen these bad results in the cases of physicians who had themselves persisted in its use for the comforting effects to their own eyes, both during the presence of foreign bodies on the cornea and after their removal.

Cocaine is contra-indicated also in any corneal inflammation, and should not be prescribed beyond the acute stage of any form of conjunctivitis.

I have had but little experience in its use beyond its local application to mucous membrane surfaces, because I have never believed that its range of utility extended any further, except perhaps, in a very few instances; hence I know very little of its general usefulness hypodermically, as it has usually seemed to me to be quite as painful to introduce it in this manner as the pain of a minor operation would be, especially when we consider the limited quantity that can be borne when it is introduced so directly into the circulation. I have sometimes, however, injected the solution into the tonsilar mass to to hasten anæsthesia, but the toxic effects follow so quickly after its introduction into the circulation in this manner, that I always hesitate to resort to this means in an untried subject. Yet, in some operations on the eye, as in tenotomies, iridectomies, cataract operations, etc., I frequently use the syringe for introducing a weak solution—one to two per cent.—subconjunctivally, in subjects that do not succumb to a four to six per cent. solution applied superficially for fifteen to twenty minutes at short intervals.

In preparing for a tonsil amputation, I use a cotton probe saturated in a five to a fifteen per cent. solution, and apply it to the whole surface of the mass once; then I send the patient to a waiting room and perhaps in the meantime—about eight or ten minutes—see another patient, then make a second thorough application, and wait three to five minutes before I use my knife and hook tonsilotome.

Occasionally, in an extremely sensitive patient who has not by this time exhibited any of the general effects, a five per cent. solution may be introduced into the tonsilar mass with a long hypodermic needle before proceeding to amputate.

In nearly five thousand tonsil amputations (four-fifths of which were done under cocaine) I have never had a dangerous hemorrhage, and not more than a dozen cases that even proved to be troublesome from this cause.

Undoubtedly, cocaine exerts a decided hæmostatic effect upon hypertrophic tissue, especially when it has thoroughly saturated the mass before an incision is made. . Yet, after the solution of continuity, its blood-staying qualities are not equal to many other hæmostatics, and it cannot be relied upon for this purpose.

During the first years of its use, this drug was vaunted by many writers in the medical journals as a great panacea—as *the sovereign remedy* for ear-ache, but, naturally, disappointment followed quickly in the practice of these theorists, who seemed not to remember that the outer layer of the membrana tympani was not a mucous layer, else they failed to remember the fact that an aqueous solution of cocaine could be only sparingly absorbed through a cutaneous epithelium.

After the rupture or incision of the drum membrane in an acute catarrhal inflammation of the middle ear, when it becomes possible for the solution to reach the membrane involved, the call for relief from pain has already ceased. When it is practical to introduce a few drops of a strong solution into the middle ear cavity through the eustachian tube, the pain is usually relieved at once; but as it is not always possible to do this on account of the swollen mouth of the tube, and as another question arises here beyond the scope of this paper, as to the advisability of the trial in any case, cocaine cannot be regarded as so great a panacea in ear-ache as it was first claimed to be by many who wrote only upon faulty theoretical grounds.

I have occasionally heard of cocaine habitues, all of whom were physicians, but I have had no personal experience with these unfortunates. Such cases will be found to be linked with the whisky habit in the great majority of instances.

I think, however, that we should always remember the danger of such a consequence when making a prescription containing cocaine, and be guarded when even its suggestion arises.

Like all drugs that are potent for good, cocaine is also potent for evil. Like fire, it is a good servant but a bad master.

